# Decompression technique — A modified approach for lateral alveolar ridge augmentation: A case report

**DOI:** 10.1002/ccr3.3746

**Published:** 2021-01-03

**Authors:** Algirdas Puisys, Viktorija Auzbikaviciute, Egle Vindasiute‐Narbute, Saulius Zukauskas, Justina Deikuviene, Dainius Razukevicius

**Affiliations:** ^1^ Vilnius Implantology Center Vilnius Lithuania; ^2^ Vilnius Research Group Vilnius Lithuania; ^3^ Vilnius University Vilnius Lithuania; ^4^ Lithuanian University of Health Science Kaunas Lithuania

**Keywords:** bone augmentation, decompression technique, guided bone regeneration, supracrestal tissue height

## Abstract

A particular technique can increase the mucosal and peri‐implant bone thickness, prevent compression around implant neck, and obtain proper space for the following prosthetic treatment with an adequate emergence profile.

## Graphical abstract

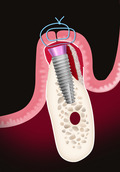

## 1 INTRODUCTION

Various bone augmentation techniques are widely used for alveolar ridge regeneration before implant placement.^1^, ^2^ Guided bone regeneration (GBR) principle is one of the most popular.^3^, ^4^ This approach provides the possibility to recover bone architecture using particulate bone graft together with a resorbable membrane as a barrier to stabilize and protect the graft.^5^ Literature suggests specific recommendations regarding different materials used for GBR.^6^ A resorbable natural collagen membrane and 1:1 mixture of autogenous bone chips with anorganic bovine bone mineral (xenograft) have been documented and recommended for horizontal augmentation.^6^, ^7^ While analyzing histological animal studies, the assumption could be made that xenograft after healing is not so homogenous in comparison with mineralized cancellous bone allograft.^8^, ^9^ It is important to investigate whether better results can be expected by changing the material used, such as from bovine bone to allogenic bone.^10^

Simultaneous implant placement can be performed with GBR if primary stability is achieved.^11^, ^12^ According to Schwarz, after bone augmentation, sufficient bone does not regenerate in the buccal site and small implant dehiscence is observed. If the buccal site of the implant is not covered, it may have a higher risk of development of peri‐implant disease or mucosal recession.^13^ One of the reasons for smaller bone regeneration volume may be an intense pressure on the implant collar from the tissues. Space maintenance is crucial for bone regeneration,^14^ therefore using a 2‐mm healing abutment instead of cover screw may shift the pressure from the implant collar to the healing abutment level and consequently, create more space for bone augmentation.

Another reason for bone resorption around the implant collar may be short supracrestal tissue height (STH).^15^ An STH of 3‐4 mm is recommended for long‐term results.^16^ Acellular dermal matrix derivative (Mucoderm, Botiss Biomaterials) was introduced for increase of supracrestal tissue height^17^, ^18^ and shows appropriate healing while simultaneously increasing STH and mucosal thickness.^19^ Thus, it is important to evaluate whether mucoderm can be used as a barrier in GBR.

This case report aims to describe decompression technique, which enables increase in bone volume by reducing compression at the implant collar and thickening of soft tissue, simultaneously with implant placement.

## 2 MATERIALS AND METHODS

The following case report was prepared according to the CARE guidelines.

### 2.1 Case presentation

A 42‐year‐old woman with a history of dentoalveolar infection and tooth loss, was referred from a colleague for lateral ridge augmentation in the posterior mandible.

**FIGURE 1:**
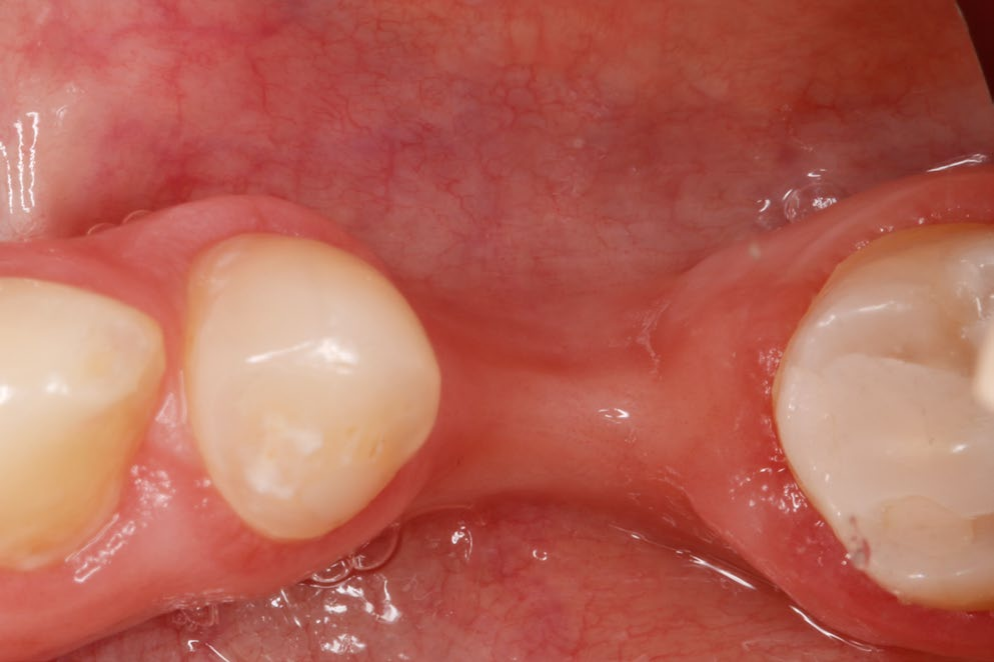
Initial intraoral condition

During intraoral observation, a horizontal ridge defect was visible at the site #36 (Figure 1).

**FIGURE 2:**
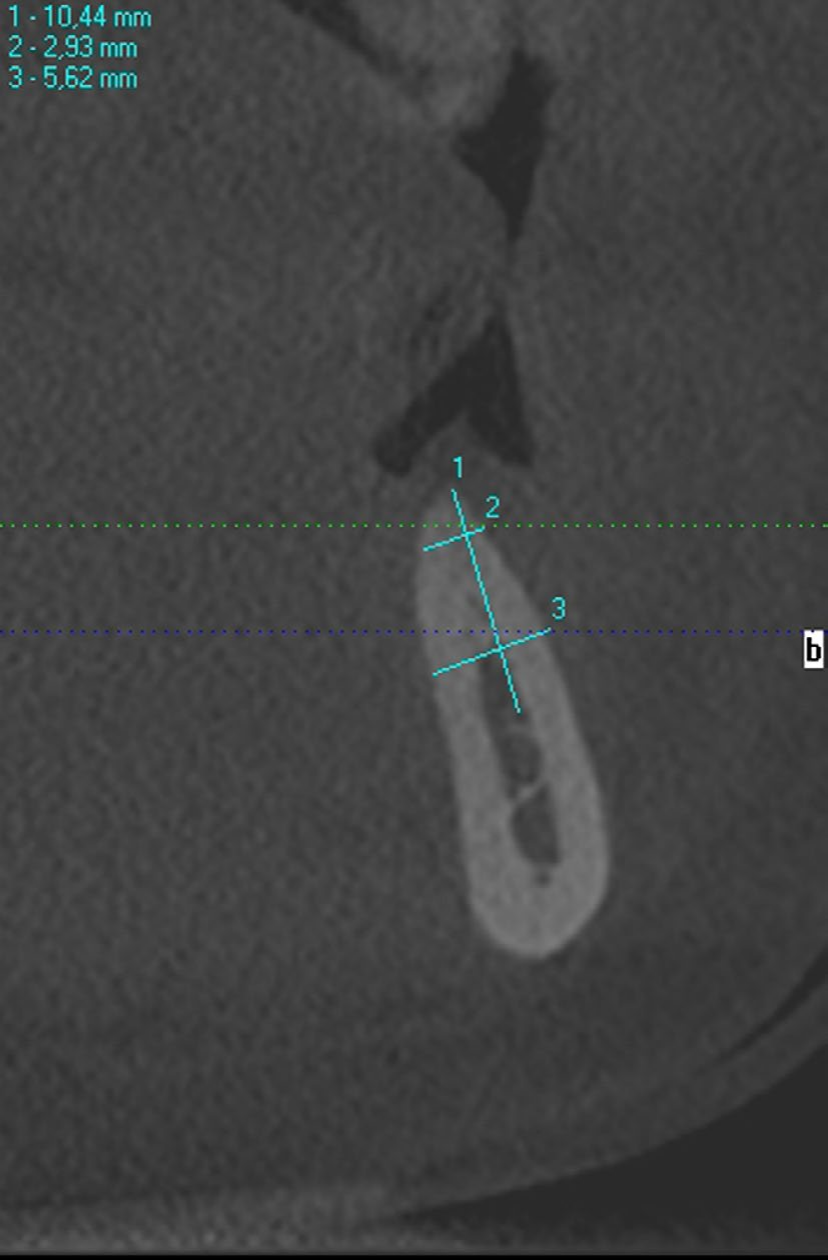
Initial condition on CBCT

A dental cone beam computed tomography (CBCT) scan was conducted in order to evaluate the present bone dimensions. As expected, a severe horizontal ridge defect was noted (class IV according to the Cawood and Howell classification).^20^ Bone width at the coronal part was estimated as 2.93 mm (Figure 2).

No medical history, oral, or systemic health problems, which might have an impact on the treatment and subsequent healing, were reported. Since the patient desired a fixed reconstruction, the clinical treatment plan was to regenerate the tissues horizontally and place the dental implant simultaneously.

### 2.2 Surgical procedure

#### 2.2.1 Stage I

The patient was premedicated with a 2‐g dose of amoxicillin (Ospamox^®^; Biochemie) an hour before the surgery. Surgery was performed under local anesthesia using 4% articaine solution with a vasoconstrictor, epinephrine (1:100 000) (Ubistesin forte^®^; 3M ESPE).

**FIGURE 3:**
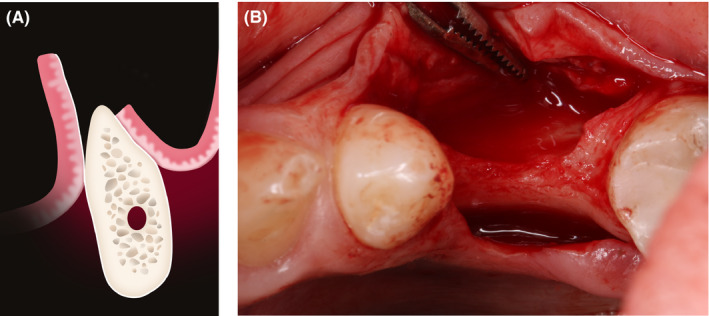
A, Horizontal incision positioned slightly buccally; (B) Full–thickness flap elevation without any vertical incisions

In the posterior mandible, a full‐thickness, slightly buccal incision was made in the keratinized gingiva using a surgical scalpel (number 15c) involving one tooth mesially (Figure 3A). For surgical access, no additional vertical incisions were needed; clear visibility of the surgical area was achieved. Then, a periosteal elevator was used to reflect a full‐thickness flap beyond the mucogingival junction (Figure 3B).

The important step is to ensure tension‐free closure. It was achieved by releasing the lingual flap with a periosteal elevator. The buccal site of the mandible was prepared by splitting the periosteum at the deepest part with the tip of a new surgical scalpel blade (15c) in the mucogingival junction area.

Thereafter, autogenous bone chips were harvested from the donor site with a cortical bone scraper (Micross^®^, META) and mixed with allogenic bone graft (Maxgraft^®^, Botiss Biomaterials) in a 1:1 proportion.

**FIGURE 4:**
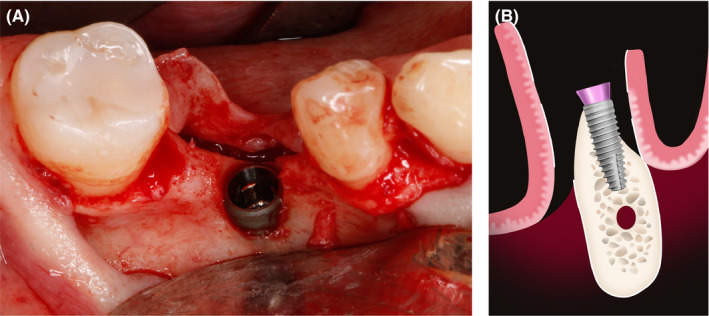
A, Exposed dental implant buccally; (B) 2 mm healing abutment

The buccal cortical bone was cleaned and inner side perforations were made to increase angiogenesis for the rate of graft incorporation. Subsequently, a 4.1 × 10 mm Straumann^®^ Bone Level Tapered Implant with SLActive surface was placed at the site #36 and good primary stability was achieved (Figure 4A). Instead of a cover screw, a 2‐mm healing abutment was used (Figure 4B). In this case, healing abutment ensured that the tension of soft tissues will be moved further from the implant neck.

**FIGURE 5:**
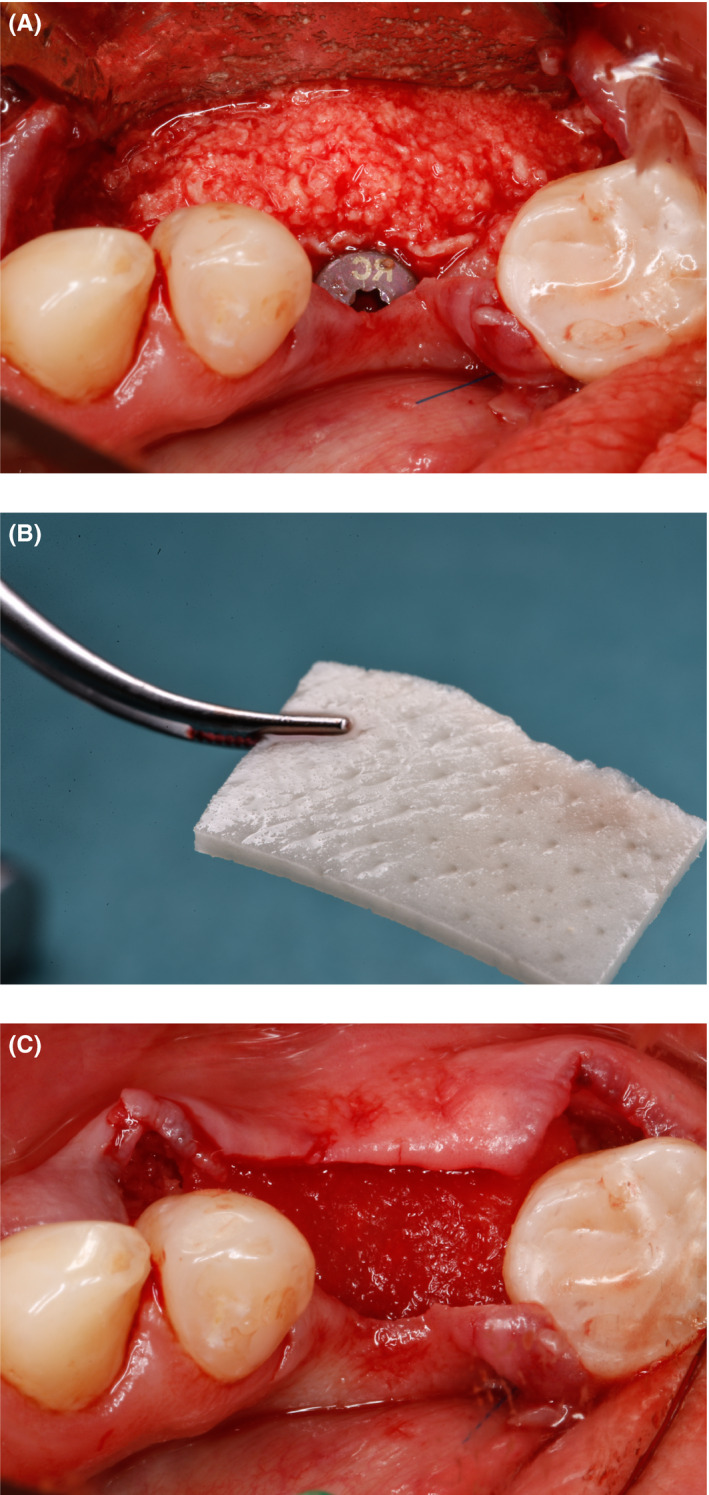
A, Bone graft of autogenous and allogenic particles placed buccally; (B) Acellular dermal matrix membrane; (C) Bone graft covered with the membrane; no pins are needed

Once the implant was placed, the prepared bone graft was positioned buccally on the defect side (Figure 5A). Subsequently, a 15 × 20 mm collagen tissue matrix derivative membrane (Mucoderm^®^, Botiss Biomaterials) (Figure 5B) was trimmed, positioned, and rehydrated with metronidazole solution for better adaptation to the augmented area, and additional prophylaxis was performed. The membrane was fixed during suturing with suture material, and no pins were needed to stabilize it. (Figure 5C).

**FIGURE 6:**
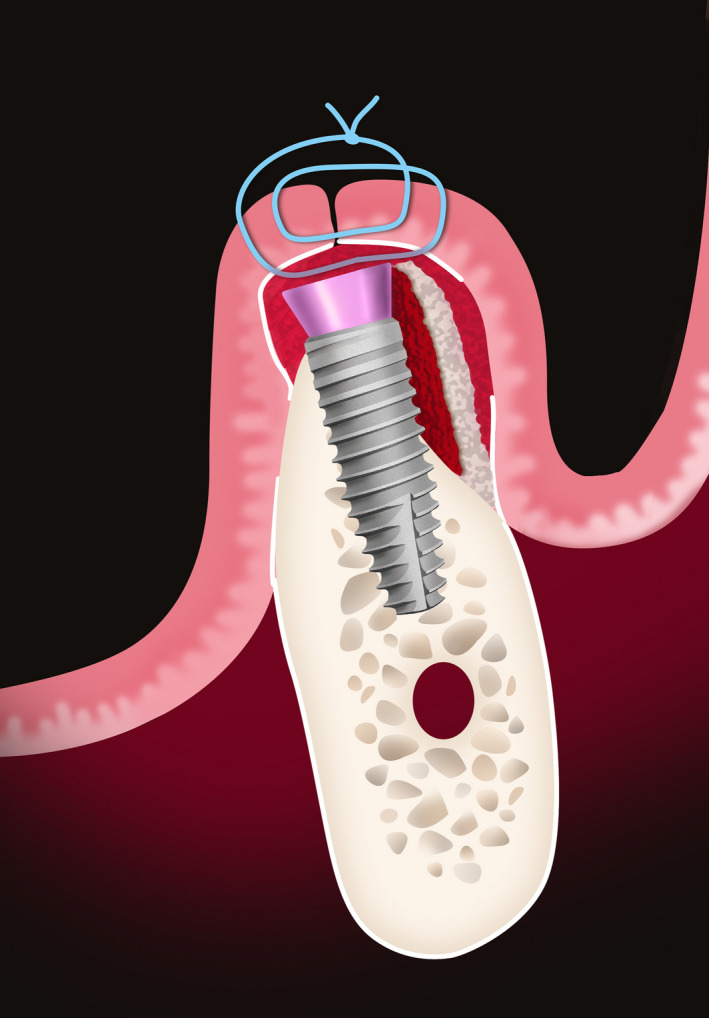
Soft tissues sutured with double simple technique

Soft tissues were sutured with double simple 6/0 sutures (Figure 6) using polypropylene suturing material. In the first round, the needle perforated all layers: buccal flap, mucoderm, and lingual flap; in the second round, only buccal and lingual flaps were perforated.

Postoperative medication included analgesics (ibuprofen), 0.12% chlorhexidine digluconate mouth rinse (Perio‐aid^®^; Dentaid) 2‐3 times a day for 1 week, and antibiotic dose (amoxicillin 1000 mg, 7 days, 2 times/day).

The patient underwent a checkup after 1 week. No extraoral swelling was observed. The patient had no complaints. Sutures were removed 2 weeks after the surgery, once the tissues were fully healed.

#### 2.2.2 Stage II

**FIGURE 7:**
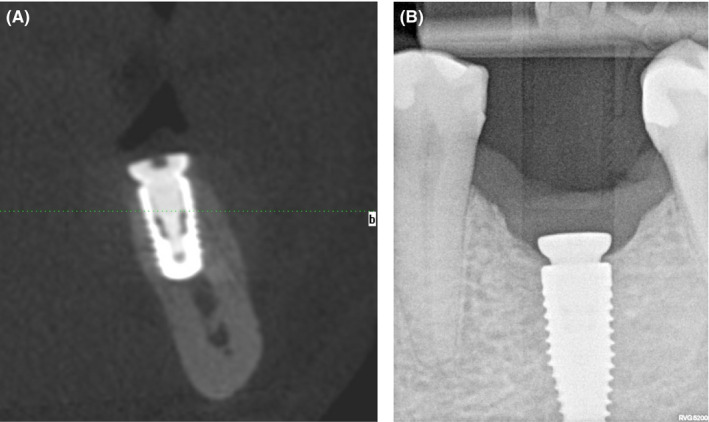
A, Cross‐section CBCT image after 6 mo; (B) Periapical radiograph after 6 mo

Six months after the stage I surgery, the patient presented for the second surgery. A new CBCT scan was performed to analyze the alveolar ridge horizontally. It was observed that the bone width increased up to 8‐mm (Figure 7A). Additional periapical radiograph showed that the crestal bone was 1‐mm above the implant neck (Figure 7B).

**FIGURE 8:**
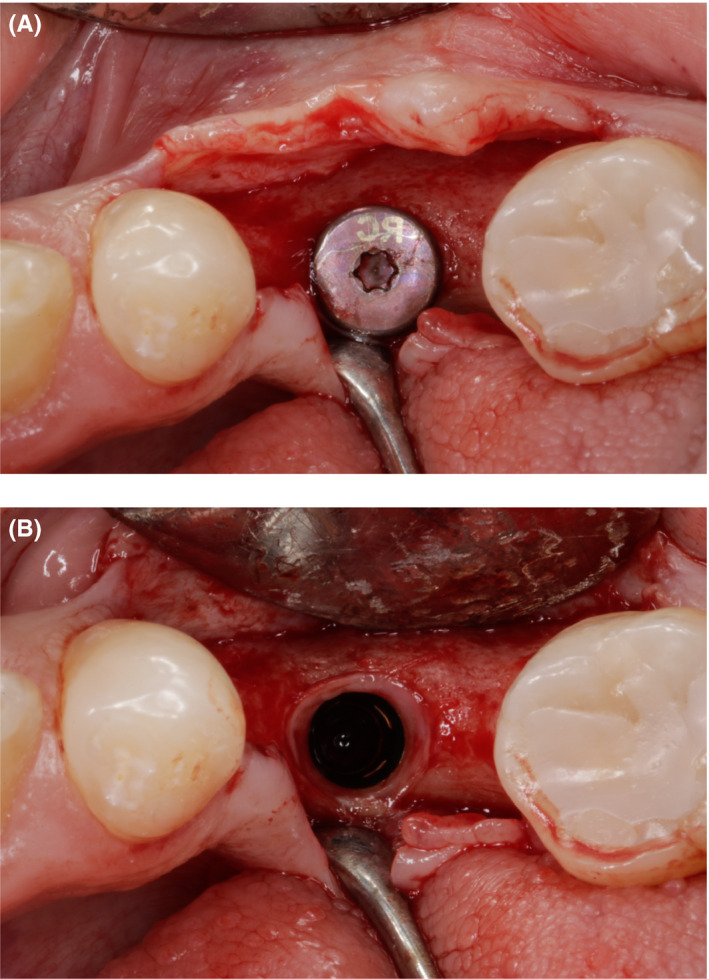
A, Increased bone width 6 mo after augmentation; (B) Bone above the implant neck and sufficient supracrestal tissue height

During the second stage, local anesthetic was administered (Ubistesin Forte^®^; 3M ESPE) and a full‐thickness flap was elevated. Homogenous bone (approximately 2 mm) around the healing abutment and 8‐mm of bone width were observed (Figure 8A). After the healing abutment was unscrewed, the bone above the implant neck and regenerated supracrestal tissue height were observed (Figure 8B). The healing abutment was replaced with a bottle neck healing abutment.

Although supracrestal tissue height was estimated to be 3 mm, it was decided to increase the thickness buccally by using a connective tissue graft from the donor site (tuberosity).

**FIGURE 9:**
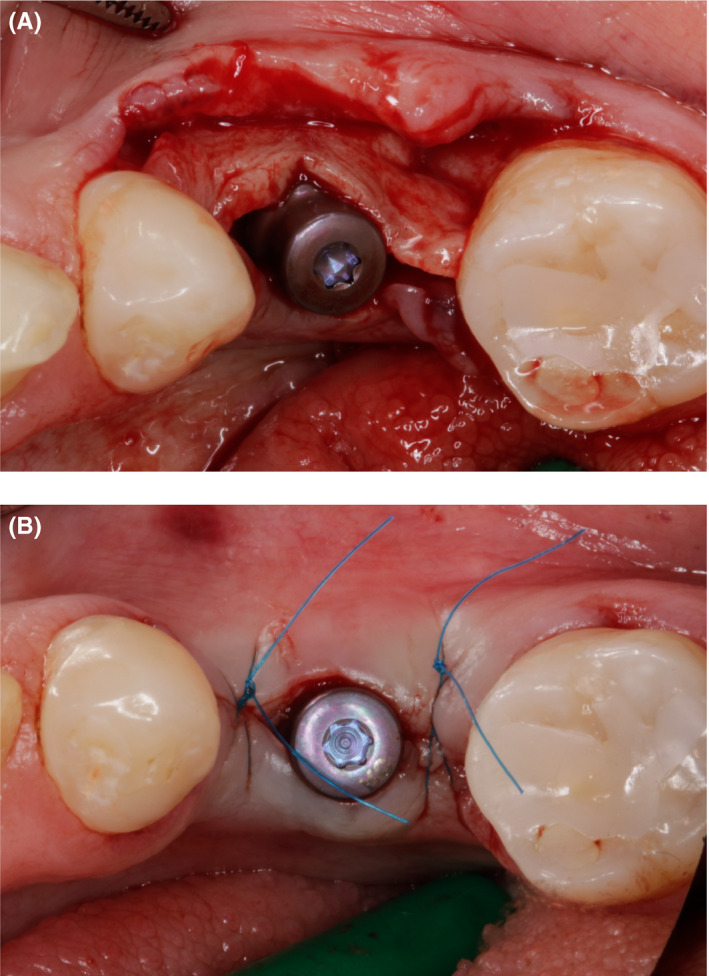
A, Connective tissue graft placed buccally; (B) Sutured soft tissues with double simple technique

Deepithelialized graft was sutured to the buccal flap, and the wound was sutured with double simple suture using 6/o polypropylene (Figure 9A, B).

### 2.3 Restorative phase

The patient underwent prosthetic treatment after 2 months when a screw‐retained zirconia crown was made. The patient underwent a regular checkup after 1 month. No bleeding on probing or suppuration were observed at the implant site (#36), and the deepest probing depth (PD) mesiolingually, mesiobuccally, distolingually, and distobuccally was measured up to 2.5‐mm.

**FIGURE 10:**
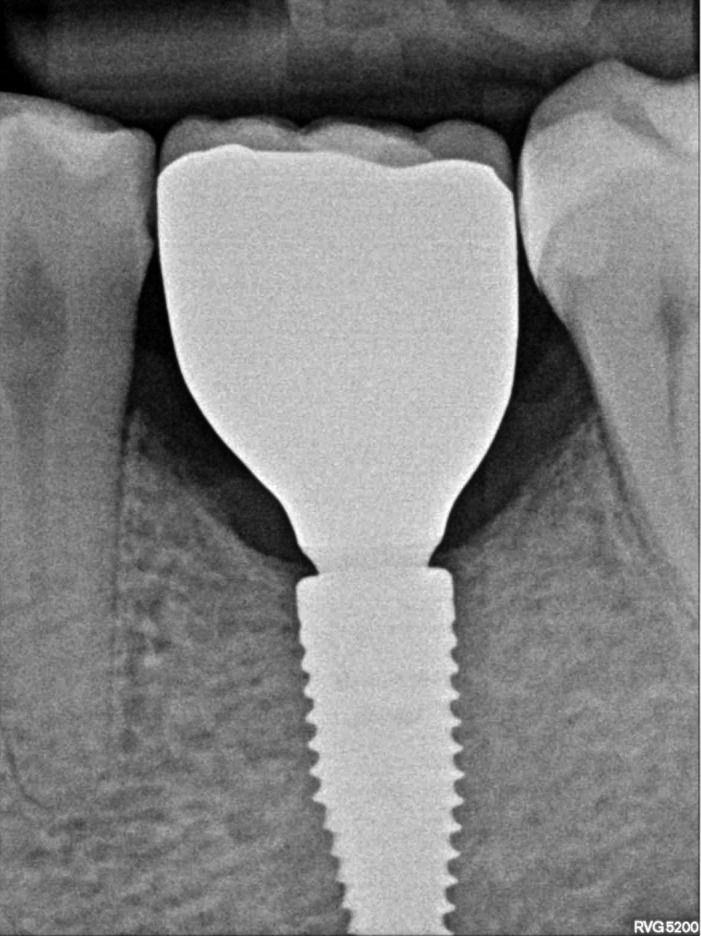
Stable crestal bone around dental implant after 4 y

**FIGURE 11:**
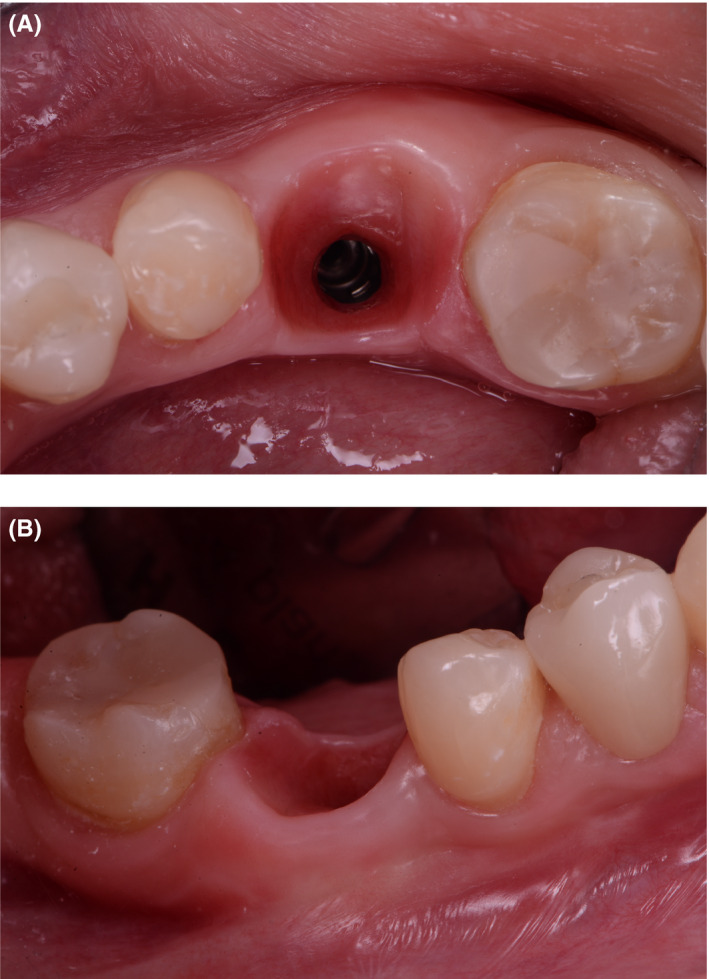
A, Postoperative occlusal view; (B) Postoperative buccal view

Clinical follow‐up after 4 years showed stable results. According to the new periapical radiograph, the crestal bone remained the same (1 mm above the implant neck) (Figure 10). During intraoral examination, no bleeding on probing was observed, and no suppuration was registered. PD at the deepest pocket was 2.5 mm (Figure 11A, B).

## 3 DISCUSSION

The present case showed successful reconstruction of hard and soft tissues using decompression technique for lateral alveolar ridge augmentation. The fundamental results are based on three small improvements: 2 mm healing abutment instead of cover screw, collagen tissue matrix derivative membrane instead of collagen membrane, and allogenic bone graft instead of xenograft.

This report clearly has some limitations. The main drawback is that we have presented one clinical case as a case report with low validity for evidence‐based results. Furthermore, as a limitation of our article it could be mentioned that the use of allogenic bone is forbidden in some countries. Moreover, the presented case report may provide only few ideas for future clinical investigations.

There are several possible adverse events of this proposed treatment method, such as exposure of the wound and the 2 mm healing abutment, especially if the implant placement was subcrestal. Suturing technique, low supracrestal tissue height, or infection may have an influence during the healing phase. Adequate flap release and precise suturing are mandatory while using this technique.

For horizontal guided bone regeneration, recommendation by several other authors is to use autogenic bone mixed with bovine bone substitutes.^21^ In this case report, it was decided to use allogenic bone chips. Both grafting substitutes have contrasting characteristics in terms of quantity and quality of the newly formed bone. El Chaar et al^8^ presented a preclinical in vivo study in minipigs that compared new bone formation around implants using mineralized cancellous bone allograft (MCBA) and sintered bovine bone mineral (SBBM). Within the MCBA group, the bone surrounding the implant appeared less dense with larger trabecular spaces than that of the SBBM group. This implies that the quantity of SBBM bone was higher than MCBA. However, the homogeneity of the new bone formed in the MCBA group was higher than that of the SBBM group. In a randomized histomorphometrical investigation by Froum et al,^9^ the same bone substitutes were compared and it was observed that the SBBM group presented higher residual bone graft material, while the MCBA group reported significantly better bone formation. Since the lower jaw is inherently denser and its blood circulation is lower, we must consider the bone quality rather than quantity.

Resorbable collagen membranes are mostly used in horizontal ridge augmentation procedures in order to stabilize the bone graft and prevent non‐osteogenic cell migration at the augmented site.^22^ In this case report, we demonstrate the possibility of replacing the graft material with a porcine‐derived collagen matrix membrane. As it can be observed, this material aids in increasing soft tissue thickness and relieving pressure on the implant neck and nearby bone. A recent case series by Puisys et al^19^ concluded that porcine‐derived collagen matrix membranes increase the soft tissue thickness by 1.8 mm on average and can be successfully used for increasing supracrestal tissue height, which was also shown through perfect integration of the membrane during histological examination. Similar results were shown in previously mentioned studies by Stefanini^17^ and Eeckhout.^18^

Manufacturers’ improvements in dental implants also have meaningful value in terms of encouraging clinical results. Many authors discussed and came to one conclusion that platform switching as well as conical implant‐abutment connection play an important role in crestal bone stability.^23^, ^24^, ^25^, ^26^, ^27^ Straight or convergent implant neck shape creates less compression on the cortical bone, thus reducing the chance of bone dehiscence.^23^ Platform switching distances the interface of the implant abutment from the bone. This feature results in more stable peri‐implant tissues and keeps the contaminated area and mechanical stress further away from the crestal bone.

The use of 2 mm healing abutment shows various advantages in comparison with a cover screw. It can be summarized that, during the one‐stage approach while using a 2‐mm healing abutment, we can achieve subcrestal implant position and augment STH.^28^, ^29^

Furthermore, during the second surgery of implant disclosure, there is no need to reflect a full‐thickness flap, especially when it is generally accepted that elevation of a periosteal flap is directly correlated with the risk of crestal bone loss.^30^, ^31^ A short incision is sufficient to replace the 2 mm healing abutment with another abutment . However, there is only one exception for the previously mentioned statement when the full‐thickness flap should be elevated, such as during surgery for augmentation of mucosal thickness using a connective tissue graft.^32^

## 4 CONCLUSIONS

In this clinical case, we achieved good clinical results, thereby, proving the possibility of decreasing crestal bone loss around the implant collar and increasing supracrestal tissue height during the one‐stage approach of lateral guided alveolar ridge augmentation. Further documentation of randomized controlled clinical trials is needed in order to address the following issues:

Whether allogenic bone is better for GBR in the lower jaw.

If the acellular dermal matrix has the same barrier function as collagen membrane and may increase supracrestal tissue height during the GBR procedure.

Whether a 2‐mm healing abutment instead of a cover screw can reduce compression on the implant neck.

Finally, if all these improvements can ensure better crestal bone as well as tissue thickness stability for a long term.

## CONFLICT OF INTEREST

None declared.

## AUTHOR CONTRIBUTIONS

AP: reviewed the literature, developed the concept, performed the procedure; VA: collected data, designed the case report; EVN: reviewed the literature and was involved in data collection; SZ: was involved in data analysis and drafted the manuscript; JD: drafted the manuscript and collected data; DR: reviewed the literature and was involved in data analysis/interpretation.

## ETHICAL APPROVAL

Informed consent has been obtained from the patient for the publication. Therefore, no additional permission from our Ethics Committee was required.

## Data Availability

This case report is used for free access.
